# Exploring Available Plastic Surgery Reward Programs and Proposing a Modeled Approach

**DOI:** 10.1093/asjof/ojae128

**Published:** 2024-12-23

**Authors:** Logan G Galbraith, Daniel Najafali, Gregory A Greco, Raman Mehrzad

## Abstract

There is a paucity of literature exploring private practice reward programs within plastic and aesthetic surgery. In this study, the authors explore private practice websites and identify universal commercial programs and practice-specific programs being adopted. They aim to evaluate the current landscape of private practice reward programs by examining their advantages and limitations. Additionally, they propose an “ideal” loyalty program that could enhance patient engagement, satisfaction, and practice growth. A review was performed to identify available reward programs and models. Private practice websites were examined for available reward models. Commercial loyalty programs were cross-referenced to determine practices leveraging them. Data collected included private practice reward program type, region, and total surgeons at the practice. Advantages and areas of improvement were analyzed of existing programs and discussions with private practice surgeons generated proposals of an ideal loyalty program model. There were no articles on plastic surgery reward programs. Private practice websites suggested 3 universal reward systems, including Allē (Allergan, Inc., Dublin, Ireland), Aspire (Galderma, Lausanne, Switzerland), and Evolus (Newport Beach, CA). A total of 4 private practices with custom reward programs were identified. Universal reward systems are point-based and often lack excitement for the consumer. These benefits accumulate points across different providers, but often lack practice-specific reward opportunities. Although beneficial, most commercially available loyalty programs do not integrate into electronic medical records (EMRs) to track usage and challenges exist in tracking patient utilization. The ideal platform harmonizes the advantages of commercial loyalty programs with tailored practice-specific rewards while having the capacity to integrate with the EMR and capture patient utilization.

**Level of Evidence: 5 (Therapeutic):**

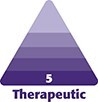

Plastic surgeons have increasingly adopted reward programs to incentivize service utilization in their practices. However, there is a notable absence of literature outlining a theoretical framework for maximizing revenue generation without compromising customer retention or care quality. Given the range of services offered at varying price points, we propose there must exist an optimally balanced approach to loyalty programs that can be adopted by current practices. Combining benefits from preexisting commercial programs, tailoring it to a given practice, and overcoming pain points will create the ideal model.

In the evolving landscape of private practice, particularly within the plastic and reconstructive surgery field, the implementation of reward programs is an underutilized strategy that holds significant potential for fostering patient loyalty. Despite the clear advantages of such programs, a surprisingly small number of practices have embraced them. McKinsey & Company notes that loyalty programs are often overlooked as an area for performance improvement and that leading loyalty programs can increase revenue by 15% to 25%.^1^ Catalogs of diverse items are also popular structures that comprise reward programs, and broad selections may likely differ in appeal due to heterogeneity.^2^ Those that do often offer only rudimentary incentives, which lack the appeal and value necessary to meaningfully engage patients. This stands in stark contrast to other industries, such as retail and hospitality, where loyalty programs have been meticulously developed not only to attract customers but also to create lasting relationships and repeat business. For instance, companies like Starbucks (Seattle, WA) have revolutionized customer engagement with their loyalty programs, offering tiered rewards that encourage frequent purchases and deeper brand loyalty.^3-6^ Similarly, airlines have mastered the art of customer retention through their frequent-flyer programs, providing a range of benefits that increase with the customer's level of engagement.^7,8^ These industries demonstrate that when designed thoughtfully, reward programs can serve as powerful tools for enhancing customer satisfaction and retention. Plastic and reconstructive surgery practices, by adopting and tailoring these more sophisticated approaches, can not only differentiate themselves in a competitive market but also build stronger, more loyal patient bases, ultimately driving long-term success. The impact of a well-tailored loyalty program is evident in practices that integrate marketing efforts with community engagement events, such as annual open houses, and innovative membership programs. For example, a practice in modestly sized Louisville, Kentucky (29th largest city with population of ∼618,000) has successfully implemented a reward program that generates significant revenue while enhancing patient satisfaction. Cultivating a win–win reward program is a powerful asset for plastic and reconstructive surgeons, as it allows for strategic alignment of services with patient incentives. These programs often include features such as crediting payments toward future medispa products and services, creating a mutually beneficial experience for both patients and the practice.^9,10^

Reward programs are commonplace across many industries and accessible to our patients. Consumers are accustomed to encountering diverse offers and opportunities to enroll in such programs.^11^ It follows logically that medicine has followed suit in offering these incentives. However, not all reward programs are equally appealing to consumers. For example, the American Express (Amex card), which may require membership fees in the case of their more exclusive and more travel-oriented rewards cards, may not attract everyone, yet it boasts a substantial user base attracted by its reward program targeting high-end consumer experiences.^12^ We have explored industries beyond plastic and reconstructive surgery to glean insights that could benefit our field.

Our investigation also sought to identify effective reward programs in private practice settings. Specifically, we examined private practice websites and relevant literature to compile various approaches. Our objective was to synthesize the best VIP reward program that can be easily replicated in any private practice.

The authors of this study aim to review and evaluate existing practice reward systems, ultimately proposing a superior model tailored for plastic and reconstructive private practices. We recognize that any successful reward program must mutually benefit both the plastic surgeon and the patient-consumer.^13^ Our goal, in essence, is to discover a reward program that achieves this mutual benefit.

## METHODS

### Literature Search: Database, Search Terms, Inclusion Criteria, and Data Extraction

PubMed was queried using the following search terms combined with Boolean operators: “plastic surgery rewards program,” “loyalty program,” “plastic surgery VIP program,” and “private practice rewards program.” Inclusion criteria were predetermined to consist of articles discussing the components and makeup of a loyalty or reward program for plastic and reconstructive surgery practices and/or medical spas (“med spas”). Studies needed to highlight the anatomy of these programs and discuss the impact on the practice. Eligible articles included editorials, letters to the editor, and original research articles. Data extraction consisted of the type of reward program offered, region, tiers, region, and comments on patient conversion or impact to the practice.

### Private Practice Data Mining

Google Search Engine (Alphabet Inc., Mountain View, CA) was leveraged to locate details of private plastic and reconstructive surgery practices and associated med spas loyalty programs. The search terms for Google included: “plastic surgery rewards program,” “loyalty program,” “plastic surgery VIP program,” and “private practice rewards program.” Inclusion criteria for the websites included for review were those that resulted in the first 2 pages of resultant websites and those that had links directly to a reward program landing page. Data were then extracted in a standardized Excel spreadsheet that was populated ahead of time with columns for private practice reward program type, region, and total surgeons at the practice. Practices were anonymized and identified by Practice # to de-identify the practice itself.

### Commercially Available Rewards and Loyalty Programs

The range of available rewards and loyalty programs was obtained through private practice websites and cross-referenced with company websites. Publicly accessible websites and industry reports were gathered to determine whether a practice is actively utilizing a commercial service. Therefore, a given practice that may have multiple reward programs was described as such.

### Discussions With Stakeholders and Synthesis of a Proposed Model

Once models were summarized and popular commercially available models were collected across practices, discussions were had with stakeholders who participated in a reward model in their practice. From these discussions, advantages and current challenges that would serve as areas for improvement for reward programs were revealed. This served as a basis for a discussion on the ideal model as well as current limitations of preexisting models.

## RESULTS

Our PubMed search in August 2024 resulted in no articles (*n* = 0, 0%) specifically discussing reward programs within plastic and reconstructive surgery practices. However, our Google search resulted in pages of resultant websites for exploration from which we evaluated the first 2 pages. Our findings of these practices are recorded in Table 1. A proposed reward model for a practice in an urban center is provided in Table 2. A reward model for a practice in a rural region that leveraged body banking is highlighted in Table 3.

**Table 1.** Summary of Private Practices and Their Current Rewards and Loyalty Programs

Private practice	Reward program	Region	Surgeon no.
1	Allē	Midwest	1
2	Allē, Aspire, Evolus	Midwest	1
3	Allē	Pacific	4
4	Allē, Aspire	Midwest	3
5	Allē	Northeast	1
6	Allē, Aspire	Midwest	2
7	Renaissance Rewards	Southeast	4
8	Allē	Northeast	3
9	Allē	Midwest	4
10	Allē	Pacific	6
11	Allē	Southeast	4
12	Allē, Aspire, CSPS Rewards	Southeast	2
13	Allē	Northeast	1
14	Coberly PlastiCentives Reward Program	Southeast	1
15	Allē	Southwest	2
16	GPS Loyalty Program	Midwest	1

**Table 2.** Proposed Reward Model for a Practice Located in an Urban Center

Membership level	Monthly fee	Benefits
Bronze	$50	5% off Botox and filler, 5% off hydrofacials, 5% off chemical peels, 5% off laser treatments, 15% off local juice bar
Silver	$75	All Bronze benefits, 10% off Botox and filler, 10% off hydrofacials, 10% off chemical peels, 10% off laser treatments, 15% off local gym membership, access to exclusive member-only events
Gold	$100	All Silver benefits, 15% off Botox and filler, 15% off hydrofacials, 15% off chemical peels, 15% off laser treatments, 30% off local nail salon services, priority scheduling for appointments
Platinum	$150	All Gold benefits, 20% off Botox and filler, 20% off hydrofacials, 20% off chemical peels, 20% off laser treatments, 25% off local car detailing, annual VIP member appreciation gift, complimentary luxury skincare product quarterly

**Table 3.** Proposed Reward Model for a Practice Located in a Rural Region Using Body Banking

Membership level	Cost	Benefits
Platinum	$950	Access to all membership benefits plus 30% off 1 product per month. One quarterly reward (varies by season). 25% off select medispa services, save 15% off surgeons' fees, additional 5% open house discount
Gold	$450	Access to all membership benefits plus 25% off 1 product per month. One quarterly reward (varies by season). 20% off select medispa services, save 10% off surgeons' fees, additional 5% open house discount
Silver	$200	Access to all membership benefits plus 25% off 1 product per month. 15% off select medispa services
Bronze	$50	Access to all membership benefits plus 20% off 1 product per month. 10% off select medispa services

### Provider Number

We documented the types of reward programs used by each practice, along with their geographic location and the number of surgical providers. To capture the volume of the private practices included in our search, we looked at provider numbers as a metric for objective analysis. We found that 11 private practices had 3 or fewer surgical providers. Only 5 private practices were made up of >3 surgical providers with the maximum number being 6 providers.

### Regions

In our study, we observed the distribution of practices across the regions of the United States. Specifically, we found that 3 practices were located in the Northeast, 6 in the Midwest, 4 in the Southeast, 1 in the Southwest, and 2 in the Pacific region. This geographic distribution provides insights into the regional variation in the adoption and utilization of reward programs within private practice settings in plastic surgery.

### Universal Programs

Our findings revealed that many practices utilize programs such as Allē (Allergan, Inc., Dublin, Ireland) and Aspire (Galderma, Lausanne, Switzerland), which are universal programs offered by manufacturers of leading cosmetic products. Thirteen practices utilized Allē. A similar reward program known as Aspire was utilized by 4 private practices, and only 1 private practice utilized Evolus (Newport Beach, CA). Additionally, we explored unique reward programs implemented by several practices that are practice specific and customized. These unique reward programs represented 4 of the private practices included in our study.

#### Allē

This reward program partners with commonly utilized aesthetic treatment brands, such as Botox (Allergan, Dublin, Ireland), Juvederm (Abbvie, North Chicago, IL), Kybella (Abbvie, North Chicago, IL), CoolSculpting (Allergan, Dublin, Ireland), Latisse (Allergan, Dublin, Ireland), and Natrelle breast implants (Allergan, Dublin, Ireland). Each of these various procedures equals a unique amount of points, which patients can then redeem for cash value toward procedures selling under the umbrella of Allē brands. For each 100 points earned, $10 can be applied toward these branded treatments. It is important to note that this reward program is used by multiple private practice plastic surgery centers as well as medical spas, and points can be earned through various providers participating in this program.^14^

#### Aspire

Aspire Rewards is Galderma's version of a reward program for their products. Those products include Dysport, Restylane, Sculptra, and Mentor breast implants (Galderma, Lausanne, Switzerland). The program works in a similar way to Allē, where various providers can offer this reward program to their consumers at multiple practices and locations. Aspire rewards also allow users to collect points for Galderma-branded treatments, which can then be redeemed for monetary value and can be applied to future procedures. The exchange rate for this program is also 100 points equaling $10.^15^

#### Evolus

This reward program is for 1 product known as Jeuveau (Evolus, Newport Beach, CA). The program was less forthcoming in how rewards are earned through consumption of their products; however, the website does state that rewards are available every 90 days. The number of points or products purchased to earn these rewards was not explicitly stated on their website.^16^

### Practice-Specific Programs

Of particular interest to the authors were the unique characteristics of the reward programs specific to the 4 private practices included in our study. These served as a case series that can be utilized by others to adopt and potentially use in their practice.

#### Practice 7

The first practice guaranteed that enrollees in the reward program would receive the lowest price on Botox and injectables, although the determination of these prices was not described. Interestingly, this practice also offered 10 free units of Botox upon signing up for the reward program. Additionally, the program included 1 free deluxe hydrofacial and dermaplaning procedure per year. Members of this reward program were also invited to exclusive VIP events, although these were not described on the website. A $100 gift card was also given to clients on their birthday.

#### Practice 12

The second practice offering a unique reward program was based on patient referral. For each patient referred for a cosmetic procedure, the primary patient received a $50 credit added to their account. This credit could be applied to injectable treatments, aesthetic services, or laser treatments. Interestingly, this reward program did not offer any incentives upon signing up nor did it provide a way for patients to earn rewards through their own service utilization.

#### Practice 14

The third practice evaluated employed a point reward system in which every $10 spent on either nonsurgical or surgical procedures would be converted into 1 point. Patients could also receive 20 points for referring friends who underwent procedures. The practice did not outline what these points could be redeemed for, but mentioned that points could also be used to receive 50% off skincare products, although the specifics of this offer were not described.

#### Practice 16

The fourth practice also utilized a point reward system but did not outline the amount required to earn each point. Instead, they described a point-to-value conversion rate, where 7500 points could be redeemed for a $25 store credit, for example. Additionally, upon signing up for this reward program, patients received $25 off a purchase of $100 or more.

## DISCUSSION

### Literature on Reward Programs

There were no articles regarding reward programs in private practice plastic surgery, despite it being a prevalent custom for practices. Although this outcome is not entirely unexpected, it highlights a significant gap in the field. One potential reason for this absence of literature is the lack of formal business training in plastic surgery residency programs.^17^ Despite the fact that private practice in plastic and aesthetic surgery often requires the application of business strategies, most plastic surgeons enter the field with little to no business education.^18^ This contrasts sharply with other industries, where individuals typically possess business degrees and extensive experience before launching their own enterprises. Interestingly, practices such as having a lawyer review a contract or negotiating are not as common in medicine as in other fields.^19^ In many business sectors, implementing a reward program is a well-established strategy, endorsed by professionals as a key method for fostering customer loyalty and driving growth. Boston Consulting Group (BCG) and other research show that there are an average of 22 loyalty program memberships in the average United States household and actively use 10.^20^ Moreover, BCG reports that some companies generate 60% of their revenue from loyalty program members. The lack of published studies on reward programs in plastic surgery may therefore reflect a broader gap in business acumen among plastic surgeons, stemming from the traditional focus of residency programs on clinical skills over business knowledge. Addressing this gap is crucial for advancing the field and enabling plastic surgeons to develop their practices with the same level of sophistication and success as seen in other industries.^21^ Modeling loyalty programs and consumer behaviors have provided valuable insights by examining how short-term promotions can influence retention.^22,23^ Belli et al found that loyalty programs enhance customer loyalty, specifically their meta-analysis found a significant positive outcome on both behavioral and attitudinal loyalty.^24^ Customer progress toward goals in a loyalty program is influenced by competing loyalty programs, indicating that geographic areas where other private practice reward programs serve as competition can play a large role in consumer decision making.^25^ Studying whether surgeons would value brand loyalty in the form of purchase quantity and repeat purchases (eg, behavioral loyalty) compared with intention to purchase or recommend to others (eg, attitudinal loyalty) is an important area of investigation that is yet to be explored extensively in the literature.^26^

### Unique Private Practice Models

Our study identified 4 private practices with existing practice-specific reward programs, but each was notably basic and lacked the lucrative appeal seen in other industries. These programs, while innovative in their own right, may not be enticing enough to deliver the compelling incentives that drive long-term customer loyalty. For example, 1 practice implemented a price lock model, offering patients the lowest price on cosmetic procedures in exchange for a monthly fee. Another practice employed a patient referral system, rewarding referring patients with modest incentives for successful referrals. The remaining practices used point-based systems, allowing patients to accumulate points that could be redeemed for monetary value. Combining these strategies may offer a superior model to all 3, which can also be improved upon by partnering with local businesses or looking to other industries for inspiration.

Although these models show some creativity, they fall short of the sophisticated strategies seen in other industries. For instance, American Express (Amex) offers a highly attractive reward program, where cardholders gain access to exclusive perks, cashback options, and points redeemable for a wide range of benefits—all of which often exceed the cost of the annual fee. Similarly, airlines and hospitality chains have perfected loyalty programs that offer tiered rewards, including upgrades, priority services, and even free flights or stays, creating a sense of exclusivity and significant value for their customers. These programs are not cheap but are rationalized by consumers because of the value added.

These examples illustrate the untapped potential for plastic surgery practices to develop more compelling and lucrative reward programs. By borrowing elements from successful models in other industries—such as bundling services, offering tiered rewards, and creating exclusive benefits—plastic surgery practices can create programs that resonate more deeply with their patient demographics, ultimately driving higher patient retention and practice growth.

### A Proposed Model for Implementation in Metropolitan Regions

Plastic surgeons in private practice can significantly enhance their reward programs by first mastering 2 fundamental business principles: understanding their target market and effectively segmenting that market. The target market consists of the specific group of patients the practice aims to attract and retain. Market segmentation further breaks down this broad group into more focused subsets based on factors, such as demographics, psychographics, and purchasing behaviors. By understanding these principles in depth, plastic surgeons can tailor their reward programs to meet the specific needs and desires of their patients, ensuring that the programs resonate and deliver real value.

To implement a successful reward program, the first step is to identify what the target market values most. For instance, patients who regularly seek procedures such as fillers, Botox, or hydrofacials may also prioritize self-care services, such as manicures and pedicures. Recognizing this overlap allows the practice to partner with local nail salons, offering exclusive benefits to reward program members—an offering that feels natural and valuable to the patient. Similarly, many patients might be health conscious, making collaborations with local health and wellness bars that offer freshly squeezed juices or smoothies a perfect fit for the reward program. These partnerships not only enhance the value of the reward program but also deepen the connection between the practice and its patients by catering to their lifestyles and preferences. On a similar note, the timing of these offers needs to be tailored as an individual who just received Botox a month ago may not desire or get use from another discount or reward on the service. Rather, having a rotating selection that makes sense with procedure timing and is enticing can be a good strategy. This is similar to a credit card that allows card holders to select a reward category based on usage or lifestyle preferences (eg, cashback on groceries, gas, etc).

Once the practice has identified the benefits that will appeal to its market segments, the next critical step is implementing the reward program effectively. This requires a sustainable model that controls costs while delivering consistent value and is one that is dynamic with changing times. One approach is to collaborate with local vendors who can submit receipts for services used by the practice's patients each month. This pay-per-use model ensures that the practice only incurs costs when rewards are redeemed, avoiding unnecessary overspending. Additionally, this model allows for flexibility; in months when fewer rewards are claimed, the practice's expenses decrease, making the program more financially manageable. By carefully balancing cost control with valuable offerings, plastic surgeons can create a reward program that not only attracts and retains patients but also supports the long-term growth and sustainability of their practice.

A reward system for private practice plastic surgeons should be more comprehensive than just offering perks from local vendors; it must also include meaningful incentives within the practice itself. A great example of a successful reward program that could be adapted to this context is the subscription-based model seen in the fitness industry, such as those used by premium gym memberships. For instance, companies like ClassPass (New York, NY) have shown how offering a set number of classes or services for a monthly fee can drive consistent engagement and customer loyalty. This model could be applied to a plastic surgery practice by offering a membership program where clients pay a monthly fee to receive a set number of Botox treatments, fillers, hydrofacials, chemical peels, lasers, or other cosmetic procedures annually. This not only ensures consistent patient engagement but also secures a steady revenue stream for the practice. Exclusivity can also be a model that could benefit certain markets, as seen with Equinox Fitness Club (Equinox Group, New York, NY) which is usually seen as a premier fitness experience that may be more costly than the typical gym membership.

However, to ensure that such a program is profitable, it is essential to have robust data analytics and financial break-even calculations in place. Data analytics can provide insights into patient behaviors, preferences, and spending patterns, allowing the practice to tailor its reward program to meet the specific needs of its market segments. For example, by analyzing how often patients opt for certain treatments, the practice can adjust the membership offerings to align with actual demand, maximizing both values for the patient and profitability for the practice. Again, a dynamic reward program that works to benefit the patient is ideal, but it should never remove preexisting rewards or be so dynamic in a fashion that would dissuade or insult clientele. Practices such as implementing expiration toward rewards not only may seem beneficial on the surface to practices but also may make patients hesitant, as deciding when to do a procedure or engage with a practice's services is not as simple of a timeline.

Financial calculations are equally crucial, particularly in determining the appropriate monthly fee for the membership program. This fee must be carefully calculated to cover the costs of the treatments and services provided while also generating a profit for the practice. A break-even analysis can help determine the minimum number of members needed to cover fixed and variable costs, ensuring that the program does not result in a financial loss. By continuously monitoring these financial metrics, the practice can adjust pricing, service offerings, or vendor partnerships as needed to maintain profitability.

Ultimately, the goal is to create a win–win scenario where the patient receives superior value through convenient, bundled services and exclusive perks, whereas the practice benefits from increased patient loyalty, steady revenue, and improved financial health. By leveraging data analytics and sound financial planning, plastic surgeons can implement a reward program that not only attracts and retains patients but also contributes to the sustainable growth of their practice. Our discussions with stakeholders revealed that many commercially available loyalty programs do not synergize with practices electronic medical records (EMRs) and would benefit from tracking utilization over time.

### A Proposed Model for Implementation in Rural Regions

The availability of vendors such as health and wellness juice bars, boutique gyms, and other local businesses naturally varies by region, which can influence how a reward program is structured. However, another innovative approach to building customer loyalty and transitioning nonsurgical patients into surgical ones involves offering discounted rates on various nonsurgical cosmetic procedures, such as Botox, fillers, and hydrofacials, in exchange for a monthly premium. This premium could be part of a “body banking” model where the funds accumulate in the patient's account over time and can later be applied toward a more significant surgical procedure when the patient is ready. This model draws inspiration from successful strategies in other industries, such as the retail and hospitality sectors, where companies like Sephora (Paris, France) and Starbucks have used similar methods to encourage repeat business and customer loyalty. For example, Starbucks' reward program allows customers to accumulate stars with each purchase, which can later be redeemed for free items or discounts. This not only drives consistent engagement but also increases the likelihood that customers will continue to choose Starbucks over competitors. Providing a banking-type solution also encourages patients to save money and can be implemented without the traditional style of interest over time leading to no additional cost to the practice itself. This can be difficult if practices do not manage the money well by having a separate account that patient deposited funds over time are saved in. This could be analogous to a renter that does not appropriately save their tenants security deposit, as a patient who deposits money in the practices rewards banking program over time should be able to withdraw funds immediately and without issue.

In the context of plastic surgery, the body banking model offers distinct advantages for both the patient and the provider. Patients benefit from immediate discounts on procedures they are already interested in while also building up a fund that can be applied toward future, more substantial procedures. This creates a strong incentive for patients to remain loyal to the practice, as their accumulated funds can be optimized with that specific provider.

For the provider, this model offers a more predictable and stable monthly revenue stream, which aids in financial planning and resource allocation. Knowing the consistent demand for nonsurgical procedures allows the practice to manage inventory and staff more efficiently, reducing waste and optimizing operations. Additionally, the accumulated funds could serve as a form of capital that the provider can use, potentially reducing the need for business loans or lines of credit, which often come with high interest rates. However, this can be a slippery slope as patient funds should not be used in an unethical fashion (eg, FTX Trading Ltd scandal or not following legislation and FDIC insurance). Never should such a model have a spike in customer withdrawals expose a hole in the reward programs account because funds are not available. Perhaps a potential solution to business liability in the amount of rewards issued that are outstanding and yet to be redeemed could be designating a nonrefundable percentage of banked funds ahead of time. Ensuring that consumer law and appropriate designations are made is paramount, as akin to informed consent, there must be clear and transparent communication of such reward models.^27^ Ethically, an unintended consequence of a reward balance may drive an individual to pursue surgery in the future based on a model that does not allow a refund. Thus, it would be critical to ensure that individuals can remove funds and do so without any financial punishments or coercion. This may be a future direction for cryptocurrencies block chain model where every transaction has a history and the encryption would assist in preventing theft of funds.^28-30^ By implementing such a model, plastic surgery practices can create a sustainable business strategy that enhances patient satisfaction and loyalty while also supporting the financial health and growth of the practice. Societal involvement on a national level and members of the aesthetic community should provide a set of guidelines that assist surgeons and their practices in constructing ethical rewards models.

### Limitations

This study is limited by its cross-sectional nature and reliance on a popular search engine's (Google) algorithm and search engine optimization of private practice web pages. Given the study's design, we did not survey or consult with specific surgeons or their practices to learn the details of their reward programs, how they may have evolved over time, or whether a new model had been adopted over a preexisting or advertised one. We aimed to mitigate this by using the most popular results that were returned by the search engine. Additionally, this study did not implement the proposed ideal model in a practice or gauge its popularity over time. This would have been beyond the intention of locating current reward models, identifying limitations, and providing suggested improvements. Future studies should look at the conversion rates of such a model and the satisfaction consumers and plastic and reconstructive surgeons have with it.

## CONCLUSIONS

Many med spas and private plastic surgery practices currently rely on universal reward programs, often based on point systems that lack integration into the practice's EMR. A future program may better serve the provider and patient by merging industry offerings with those unique offerings of a practice while integrating into the EMR. Although these universal systems allow patients to accumulate points across multiple providers, they may be better harmonized through customization and practice-specific offerings. To build deeper patient loyalty, we strongly recommend that a new platform emerges which improves upon those best components by combining preexisting benefits with more tailored additions that are designed specifically for an individual practice. Taking into account clientele, expertise, and overall longevity of the practice are some critical features that would be optimal in a rollout of a reward program. Our review of various private practice websites revealed that most existing programs follow a one-size-fits-all model that can actually become more innovative by adopting strategies employed in other industries. Instead of relying on such models that may not be updated over time, practices should consider implementing more dynamic reward programs—such as membership models that offer monthly procedure discounts combined with exclusive vendor perks, or a “surgical banking” plan where patients make monthly payments that accumulate toward future surgical procedures in an ethical fashion. These approaches not only enhance patient engagement and satisfaction but also create a more predictable revenue stream and foster long-term loyalty. However, to implement such programs effectively, it is crucial for plastic surgeons to acquire a solid foundation in business principles. Understanding key concepts such as market segmentation, financial break-even analysis, marketing, and data-driven decision making is essential for designing reward programs that are both profitable and appealing to patients. By integrating business knowledge into their practice, plastic surgeons can develop innovative, effective reward systems that not only meet the needs of their patients but also support the sustainable growth of their practice.
